# A review of experimental investigations on thermal phenomena in nanofluids

**DOI:** 10.1186/1556-276X-6-377

**Published:** 2011-05-09

**Authors:** Shijo Thomas, Choondal Balakrishna Panicker Sobhan

**Affiliations:** 1 School of Nano Science and Technology, NIT Calicut, Kerala, India

## Abstract

Nanoparticle suspensions (nanofluids) have been recommended as a promising option for various engineering applications, due to the observed enhancement of thermophysical properties and improvement in the effectiveness of thermal phenomena. A number of investigations have been reported in the recent past, in order to quantify the thermo-fluidic behavior of nanofluids. This review is focused on examining and comparing the measurements of convective heat transfer and phase change in nanofluids, with an emphasis on the experimental techniques employed to measure the effective thermal conductivity, as well as to characterize the thermal performance of systems involving nanofluids.

## Introduction

The modern trends in process intensification and device miniaturization have resulted in the quest for effective heat dissipation methods from microelectronic systems and packages, owing to the increased fluxes and the stringent limits in operating temperatures. Conventional methods of heat removal have been found rather inadequate to deal with such high intensities of heat fluxes. A number of studies have been reported in the recent past, on the heat transfer characteristics of suspensions of particulate solids in liquids, which are expected to be cooling fluids of enhanced capabilities, due to the much higher thermal conductivities of the suspended solid particles, compared to the base liquids. However, most of the earlier studies were focused on suspensions of millimeter or micron sized particles, which, although showed some enhancement in the cooling behavior, also exhibited problems such as sedimentation and clogging. The gravity of these problems has been more significant in systems using mini or micro-channels.

A much more recent introduction into the domain of enhanced-property cooling fluids has been that of nanoparticle suspensions or nanofluids. Advances in nanotechnology have made it possible to synthesize particles in the size range of a few nanometers. These particles when suspended in common heat transfer fluids, produce the new category of fluids termed nanofluids. The observed advantages of nanofluids over heat transfer fluids with micron sized particles include better stability and lower penalty on pressure drop, along with reduced pipe wall abrasion, on top of higher effective thermal conductivity.

It has been observed by various investigators that the suspension of nanoparticles in base fluids show anomalous enhancements in various thermophysical properties, which become increasingly helpful in making their use as cooling fluids more effective [1-4]. While the reasons for the anomalous enhancements in the effective properties of the suspensions have been under investigation using fundamental theoretical models such as molecular dynamics simulations [5,6], the practical application of nanofluids for developing cooling solutions, especially in miniature domains have already been undertaken extensively and effectively [7,8]. Quantitative analysis of the heat transfer capabilities of nanofluids based on experimental methods has been a topic of current interest. The present article attempts to review the various experimental techniques used to quantify the thermal conductivity, as well as to investigate and characterize thermal phenomena in nanofluids. Different measurement techniques for thermal conductivity are reviewed, and extensive discussions are presented on the characterization of thermal phenomena such as forced and free convection heat transfer, circulation in liquid loops, boiling and two phase flow in nanofluids, in the sections to follow.

## Thermal conductivity

The techniques employed for measurement of thermal conductivity can be broadly classified into transient and steady state methods. The transient measurement techniques frequently used are the hot wire method, the hot strip method, the temperature oscillation method and the 3ω method. Steady-state measurement using a 'cut-bar apparatus' has also been reported. These methods are reviewed below.

## The short hot wire (SHW) method

The transient short hot wire (SHW) method used to measure the thermal conductivity and thermal diffusivity of nanofluids has been described by Xie et al. [9,10]. The technique is based on the comparison of experimental data with a numerical solution of the two-dimensional transient heat conduction applied to a short wire with the same length-to-diameter ratio and boundary conditions as in the experimental setup.

**Figure 1:**
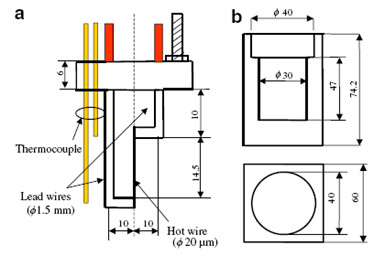
**Short hot wire probe apparatus of Xie et al. **[9].

The experimental apparatus consists of a SHW probe and a teflon cell of 30 cm^3 ^volume. The dimensions of the SHW probe are shown in Figure 1. The SHW probe is mounted on the teflon cap of the cell. A short platinum wire of length 14.5 mm and 20 μm diameter is welded at both ends to platinum lead wires of 1.5 mm in diameter. The platinum probe is coated with a thin layer (1 μm) of alumina for insulation, thus preventing electrical leakage. Before and after the application of the Al_2_O_3 _film coating, the effective length and radius of the hot wire and the thickness of the Al_2_O_3 _insulation film are calibrated. Figure 1b shows the dimensions of the Teflon cell used for measurements in nanofluids. Two thermocouples located at the same height, at the upper and lower welding spots of the hot wire and lead wires, respectively, monitor the temperature homogeneity. The temperature fluctuations are minimized by placing the hot wire cell in a thermostatic bath at the measurement temperature.

In the calculation method, the dimensionless volume-averaged temperature rise of the hot wire, θ_v _[= (*T*_v _- *T*_i_)/(*q*_v_*r*^2^/λ)] is approximated by a linear equation in terms of the logarithm of the Fourier number *Fo *[=α*t*/*r*^2^], where *T*_i _and *T*_v _are the initial liquid temperature and volume averaged hot-wire temperature, *q*_v _the heat rate generated per unit volume, *r *the radius of the SHW, *t *is the time, and λ and α the thermal conductivity and the thermal diffusivity of liquid, respectively. The coefficients of the linear equation, *A *and *B*, are determined by the least squares method for a range of Fourier numbers corresponding to the measuring period. The measured temperature rise of the wire Δ*T*_v _[=*T*_v _- *T*_i_] is also approximated by a linear equation with coefficients *a *and *b*, determined by the least square method for the time range before onset of natural convection. Thermal conductivity (λ) and thermal diffusivity (α) of nanofluids are obtained as λ = (*VI*/π*l*)(*A*/*a*) and α = *r*^2 ^exp[(*b*/*a*) - (*B*/*A*)], where *l *is the length of the hotwire, and *V *and *I *are the voltage and current supplied to the wire. The uncertainties of the thermal conductivity and thermal diffusivity measurements using SHW have been estimated to be within 1.0 and 5.0%, respectively.

## Temperature oscillation technique

**Figure 2:**
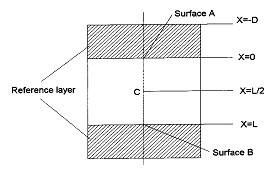
**The fluid volume for analysis corresponding to the experimental setup of Das et al. **[11].

Das et al. [11] proposed and demonstrated the temperature oscillation method for estimating thermal conductivity and thermal diffusivity of nanofluids. The method can be understood with the help of Figure 2, which shows a cylindrical fluid volume analyzed, with periodic temperature oscillations applied at surfaces *A *and *B*. The temperature oscillations are generated using Peltier elements attached to reference layer. The Peltier elements are powered by a DC power source. The real measurable phase shift and amplitude ratio of temperature oscillation can be expressed as,(1)

and(2)

where *G *is the phase shift, *u *amplitude in Kelvin, and *L *thickness of fluid sample in meter.

The complex amplitude ratio between the mid-point of the specimen and the surface can be given by(3)

where α is the thermal diffusivity and the angular velocity, ω, is given by(4)

The phase and amplitude of temperature oscillation at the two surfaces as well as at the central point *C*, gives the thermal diffusivity of the fluid, from Equations 1 or 2.

The temperature oscillation in the reference layer at the two boundaries of the test fluid yields the thermal conductivity. The frequency of temperature oscillation in the reference layer, in the Peltier element and that in the test fluid are the same.

The complex amplitude ratio at *x *= -*D *(*D *being the thickness of the reference layer) and *x *= 0 is given by(5)

where  and . The subscript R represents the reference layer.(6)

where λ is the thermal conductivity of the fluid.

The real phase shift and amplitude attenuation of the reference layer is given by(7)(8)

The thermal diffusivity of the reference layer being known either from Equations 7 or 8, the thermal conductivity of the specimen can be evaluated from Equation 6.

**Figure 3:**
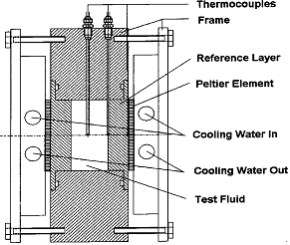
**Construction of the test cell used by Das et al. **[11].

The test cell is a flat cylindrical cell as shown in Figure 3, which is cooled on both of the ends using a thermostatic bath. DC power is applied to the Peltier element. A number of thermocouples measure the temperatures in the test section which are amplified, filtered, and fed to the data acquisition system. The frame of the cell is made of POM (polyoxymethylene), which acts as the first layer of insulation. The frame has a 40-mm diameter cavity to hold the test fluid. Two disk type reference materials of 40 mm diameter and 15 mm thickness are kept on top and bottom side of the cavity. The space for the test fluid has a dimension of 40 mm diameter and 8 mm thickness. The fluid is filled through a small hole in the body of the cell. Temperatures are measured at the interface of the Peltier element and the reference layer, at the interface of the reference layer and test fluid and the central axial plane of the test fluid. The thermocouples are held precisely centralized. The entire cell is externally insulated. The experimental setup was calibrated by measuring the thermal diffusivity of demineralized and distilled water over the temperature range of 20 to 50°C. The results showed that the average deviation of thermal diffusivity from the standard values was 2.7%. As the range of enhancement in thermal conductivity values of nanofluids is 2 to 36%, this ranges of accuracy was found to be acceptable.

## 3ω method

**Figure 4:**
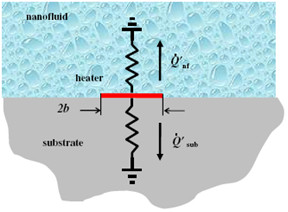
**Schematic of the experimental setup for the 3ω method reported by Oh et al. **[12].

The 3-Omega method [12] used for measuring the thermal conductivity of nanofluids is a transient method. The device fabricated using micro electro-mechanical systems (MEMS) technique can measure the thermal conductivity of the nanofluid with a single droplet of the sample fluid. Figure 4 shows the nanofluid on a quartz substrate, which is modeled as a thermal resistance between the heater and he ambient. The total heat generated from the heater (*Q*_total_) passes through either the nanofluid layer (*Q*_nf_) or the substrate (*Q*_sub_). The fluid-substrate interface resistance is neglected when the thermal diffusivities of the fluid and the substrate are similar. If Δ*T*_h _is the measured temperature oscillation of the heater in the presence of the nanofluid it can be shown that(9)

The relationship between the temperature oscillation and the heat generation rate can be expressed as,(10)(11)

where *Q*' is the heating power per unit length generated at the metal heater, *k *the thermal conductivity of the substrate, *q *the complex thermal wave number, ω the angular frequency of the input current, and ρ and *C*_p _the substrate density and heat capacity, respectively.

The temperature oscillation and the heat generation per unit heater length are related through Equation 10. It follows that a simple relationship between the temperature oscillations can be obtained as follows:(12)

Δ*T*_sub _is the heater temperature oscillation due to the heat transfer in the quartz substrate alone (measured in vacuum). The nanofluid thermal conductivity *k*_nf _is obtained from a least squares fit of Δ*T*_nf _calculated from Equation 10.

## Microlitre hot strip devices for thermal characterization of nanofluids

**Figure 5:**
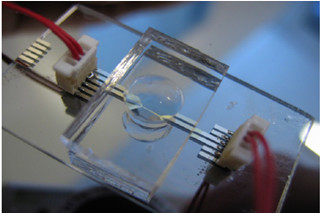
**THS device, with a water droplet confined in the open hole, as reported in **[13].

A simple device based on the transient hot strip (THS) method used for the investigations of nanofluids of volumes as small as 20 μL is reported in the literature by Casquillas et al. [13]. In this method, when the strip, in contact with a fluid of interest is heated up by a constant current, the temperature rise of the strip is monitored. Photolithography patterning of the strip was done using AZ5214 Shipley resist spin coated on a glass substrate. Electron beam evaporation deposition of Cr (5 nm)/Pt (50 nm)/Cr (5 nm) sandwich layer was followed by deposition of SiO_2 _(200 nm) cover layer deposition by PECVD (plasma enhanced chemical vapor deposition). The electrical contact areas of the sample were obtained by photolithography and reactive ion etching of SiO_2 _layer with SF6 plasma, followed by chromium etching. The micro-reservoir for nanofluids was fabricated by soft lithography. The PDMS (polydimethylsiloxane) cover block was created from a 10:1 mixture of PDMS-curing agent. The PDMS was degassed at room temperature for 2 h and cured at 80°C for 3 h. A PDMS block of 20 mm long, 10 mm large, and 3 mm thick was cut and a 5 mm diameter hole was drilled in the center for liquid handling. The PDMS block and the glass substrates were exposed to O_2 _plasma, before the device was baked at 80°C for 3 h for irreversible bonding. THS device, with a water droplet confined in the open hole is shown in Figure 5. The current and voltage measurements were performed using a voltmeter (Agilent 34410A) and a function generator (Agilent 33220A) linked to a current source. The temperature variation of the strip was recorded by applying a constant current and monitoring the resistivity change with time from which the liquid thermal conductivity was deduced.

The transient response of the platinum strip temperature can be described by the following expression for *t *> 0.2 s:(13)

where *T*_o _is the intercept on the temperature axis of the *T *vs. ln(*t*) graph. The thermal diffusivity, α_f _depends on the thermal conductivity *k*, the density, and the specific heat capacity of the fluid. As a first-order approximation, it is possible to obtain the thermal conductivity from the measurement of α_f_.

## Steady state measurement using cut-bar apparatus

**Figure 6:**
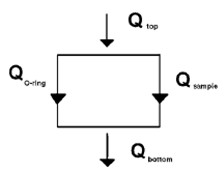
**Heat flux paths in the steady-state measurement method reported in Sobhan et al. **[14].

**Figure 7:**
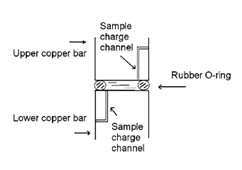
**Test cell for steady-state measurement of thermal conductivity of nanofluids **[14].

Steady-state measurement of the thermal conductivity of nanofluids using a cut-bar apparatus has been reported by Sobhan and Peterson [14]. The steady state thermal conductivity of the nanofluid can be modeled as shown in Figure 6. The apparatus consists of a pair of copper rods (2.54 cm diameter) separated by an O-ring to form the test cell as shown in Figure 7. Several thermocouples are soldered into the copper bars to measure surface temperatures and the heat flux. The test cell is placed in a vacuum chamber maintained at less than 0.15 Torr. The external convection and/or radiation losses are thus minimized, and hence neglected. The size of the test cell is kept small, such that convection currents do not set in, as indicated by an estimation of the Rayleigh number. The heat flux in the cut-bar apparatus is the average of the heat fluxes from Equation 14 below, calculated from the temperature differences between the upper and lower copper bars:(14)

where *q *is the heat flux, *k*_copper _the thermal conductivity of copper bars, Δ*T*_bar _the temperature difference along the copper bars, and Δ*Z*_bar _the distance along the copper bars.

The effective thermal conductivity of the nanoparticle suspension contained in the test cell can be calculated as:(15)

where *k*_eff _is the effective thermal conductivity of the nanofluid, *q *the heat flux, Δ*T*_cell _the average temperature difference between the two surfaces of the test cell, Δ*Z*_cell _the distance between the two cell surfaces, *k*_O-ring _the thermal conductivity of the rubber O-ring, *A*_O-ring _the cross-sectional area of the rubber O-ring, and *A*_cell _the cross-sectional area of the test cell. Baseline experiments using ethylene glycol and distilled water showed an accuracy of measurement within +/-2.5%.

## Comparison of thermal conductivity results

The transient hot wire (THW) method for estimating experimentally the thermal conductivity of solids and fluids is found to be the most accurate and reliable technique, among the methods discussed in the previous sections. Most of the thermal conductivity measurements in nanofluids reported in the literature have been conducted using the transient hot wire method. The temperature oscillation method helps in estimating the temperature dependent thermal conductivity of nanofluids. The steady-state method has the difficulty that steady-state conditions have to be attained while performing the measurements. A comparison of the thermal conductivity values of nanofluids obtained by various measurement methods and reported in literature is shown in Table 1.

**Table 1:** Thermal conductivity values

**Sl.no**.	Base fluid	Nanoparticle	Avg particle size (nm)	Conc. (vol.%)	Sonication time (h)	Temp. (°C)	Enhancement	Method of measurement	Uncertainty %
1	Distilled water	Al_2_O_3_	36	10	3	27.5-34.7	1.3 times	Steady state	2.5
2	Distilled water	CuO	29	6	3	34	1.52 times	Steady state	2.5
3	Distilled water	Al_2_O_3_	28.6	1	12	21-51	2-10.8%	Temperature oscillation	2.7
4	Distilled water	Al_2_O_3_	28.6	4	12	21-51	9.4-24.3%	Temperature oscillation	2.7
5	Distilled water	CuO	38.4	1	12	21-51	6.5-29%	Temperature oscillation	2.7
6	Distilled water	CuO	38.4	1	12	21-51	14-36%	Temperature oscillation	2.7
7	Distilled water	Al_2_O_3_	20	1	NA	5-50	10%	SHW method	1
8	Distilled water	Al_2_O_3_	45	1	15	NA	4.4%	3ω method	NA

Comparison of thermal conductivity values obtained using transient and steady-state measurement techniques.

## Viscosity

Viscosity, like thermal conductivity, influences the heat transfer behaviour of cooling fluids. Nanofluids are preferred as cooling fluids because of their improved heat removal capabilities. Since most of the cooling methods used involve forced circulation of the coolant, modification of properties of fluids which can result in an increased pumping power requirement could be critical. Hence, viscosity of the nanofluid, which influences the pumping power requirements in circulating loops, requires a close examination. Investigations [3,4,15-22] reported in the literature have shown that the viscosity of base fluids increases with the addition of nanoparticles.

Praveen et al. [15] measured the viscosity of copper oxide nanoparticles dispersed in ethylene glycol and water. An LV DV-II+ Brookfield programmable viscometer was used for the viscosity measurement. The copper oxide nanoparticles with an average diameter of 29 nm and a particle density of 6.3 g/cc were dispersed in a 60:40 (by weight) ethylene glycol and water mixture, to prepare nanofluids with different volume concentrations (1, 2, 3, 4, 5, and 6.12%). The viscosity measurements were carried out in the temperature range of -35 to 50°C. The variation of the shear stress with shear strain was found to be linear for a 6.12% concentration of the nanofluid at -35°C, which confirmed that the fluid has a Newtonian behavior. At all concentrations, the viscosity value was found to be decreasing with an increase in the temperature and a decrease in concentration of the nanoparticles. The suspension with 6.12% concentration gave an absolute viscosity of around 420 centi-Poise at -35°C.

Nguyen et al. [3] measured the temperature and particle size dependent viscosity of Al_2_O_3_-water and CuO-water nanofluids. The average particle sizes of the samples of Al_2_O_3 _nanoparticles were 36 and 47 nm, and that of CuO nanoparticles was 29 nm. The viscosity was measured using a ViscoLab450 Viscometer (Cambridge Applied Systems, Massachusetts, USA). The apparatus measured viscosity of fluids based on the couette flow created by the rotary motion of a cylindrical piston inside a cylindrical chamber. The viscometer was having an accuracy and repeatability of ±1 and ±0.8%, respectively, in the range of 0 to 20 centi-Poise. The dynamic viscosities of nanofluids were measured for fluid temperatures ranging from 22 to 75°C, and particle volume fractions varying from 1 to 9.4%. Both Al_2_O_3_-water and CuO-water nanofluids showed an increase in the viscosity with an increase in the particle concentration, the largest increase being for the CuO-water nanofluid. The alumina particles with 47 nm were found to enhance viscosity more than the 36 nm nanoparticles. At 12% volume fraction, the 47-nm particles were found to enhance the viscosity 5.25 times, against a 3% increase by the 36-nm particles. The increase in the viscosity with respect to the particle volume fraction has been interpreted as due to the influence on the internal shear stress in the fluid. The increase in temperature has shown to decrease the viscosities for all nanofluids, which can be attributed to the decrease in inter-particle and inter-molecular adhesive forces. An interesting observation during viscosity measurements at higher temperatures was the hysteresis behaviour in nanofluids. It was observed that certain critical temperature exists, beyond which, on cooling down the nanofluid from a heated condition, it would not trace the same viscosity curve corresponding to the heating part of the cycle. This was interpreted as due to the thermal degradation of the surfactants at higher temperatures which would result in agglomeration of the particles. A comparison of the viscosity values of nanofluids reported in literature [3,4,15-22] is shown in Table 2.

**Table 2:** Viscosity values

**Sl.no**.	Reference	Nanoparticle used	Basefluid	Concentration	Temp range	Percentage enhancement in viscosity
1	Praveen et al. [15]	CuO (29 nm)	60:40 (in weight) ethylene glycol and water mixture	1, 2, 3, 4, 5, 6.12%	-35 to 50°C	For 6.12% conc: 4.5 times @ 35°C and 3 times @ 50°C
2	Nguyen et al. [3,16]	CuO (29 nm) Al_2_O_3_(36 and 47 nm)	Water	1-12%	22 to 75°C	CuO @ 9%: 7-10 times Al_2_O_3_(36 nm) @ 9%: 4.5-3.5 times Al_2_O_3_(47 nm) @ 9%: 5.4-4.4 times
3	Chen et al. [17]	Titanate nanotubes (diameter approx. 10 nm, length approx. 100 nm, aspect ratio approx. 10)	Ethylene glycol	0.5, 1.0, 2.0, 4.0, and 8.0% by weight	20-60°C	@ 8%: High shear viscosity is in the range of 10-35 m Pa s
4	Phuoc et al. [18]	Fe_2_O_3 _(20-40 nm)	Deionized water containing 0.2% polymer by weight as a dispersant.	1, 2, 3, 4%	25°C	@ 2%: Infinite viscosity is 12.25 cP for 0.2% PEO (Polyethylene oxide) surfactant, and 2.58 cP for 0.2% PVP (Polyvinylpyrrolidone) surfactant
5	Garg et al. [19]	MWCNT (multi-walled carbon nanotube) (diameter of 10-20 nm, length of 0.5-40 μm)	Deionized water with 0.25% by mass of gum Arabic	1% by mass	15 and 30°C	Viscosity of nanofluids increases with sonication time. Beyond a critical sonication time it decreases due to increased breakage of CNTs
6	Murshed et al. [20]	TiO_2 _(15 nm)/Al_2_O_3 _(80 nm)	Deionized water with Cetyl Trimethyl Ammonium Bromide (CTAB) surfactant (0.1 mM)	1-5% by volume	-	@ 5% of Al_2_O_3 _viscosity increases by 82% @ 4% of TiO_2 _viscosity increases by 82%
7	Chena et al. [21]	TiO_2 _(25 nm) and TNT (Titanate nanotubes) (diameter approx. 10 nm, length approx. 100 nm, aspect ratio approx. 10)	Water, ethylene glycol	0.1-1.8% by volume	-	@ 0.6% of water-TNT 80% increase in viscosity @ 1.8% EG-TNT 70% increase in viscosity @1.8% EG-TiO_2 _20% increase in viscosity
8	Duangthongsuk et al. [4]	TiO_2 _(21 nm)	Water	0.2, 0.6, 1.0, 1.5, and 2.0% with pH values of 7.5, 7.1, 7.0, 6.8, and 6.5,	15, 25 and 30°C	@ 15°C for the conc. range of 0.2-2% viscosity increases by 4-15%.
9	Lee et al. [22]	Al_2_O_3 _(30 ± 5 nm)	Deionized water (DI)	0.01-0.3 vol.%	21-39°C	@ 21°C for the conc. range of 0.01-0.3% viscosity is enhanced by 0.08-2.9%

Comparison of viscosity enhancement in various nanofluids.

## Forced convection in nanofluids

Forced convection heat transfer is one of the most widely investigated thermal phenomena in nanofluids [23-35], relevant to a number of engineering applications. Due to the observed improvement in the thermal conductivity, nanofluids are expected to provide enhanced convective heat transfer coefficients in convection. However, as the suspension of nanoparticles in the base fluids affect the thermophysical properties other than thermal conductivity also, such as the viscosity and the thermal capacity, quantification of the influence of nanoparticles on the heat transfer performance is essentially required. As the physical mechanisms by which the flow is set up in forced convection and natural convection are different, it is also required to investigate into the two scenarios individually. The case of the natural convection (thermosyphon) loops is another problem in itself, because the characteristic of the flow is similar to that of the forced convection loop, though the mechanism is buoyancy drive. Some of the important investigations on forced convection in nanofluids are reviewed in this section. Studies on free convection and thermosyphon loops will be discussed in the sections to follow.

## Convective heat transfer in fully developed laminar flow

**Figure 8:**
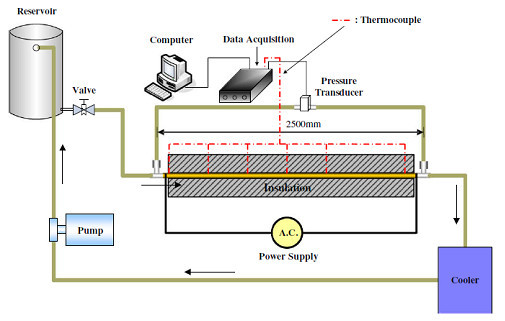
**Experimental setup of Hwang et al. **[23].

Experimental investigations on the convective heat transfer coefficient of water-Al_2_O_3 _nanofluids in fully developed laminar flow regime have been reported by Hwang et al. [23]. Their experimental setup consisted of a circular tube of diameter 1.812 mm and length 2500 mm, with a test section having an externally insulated electrical heater supplying a constant surface heat flux (5000 W/m^2^), a pump, a reservoir tank, and a cooler, as shown in Figure 8. T-type thermocouples were used to measure the tube wall temperatures, *T*_s_(*x*), and the mean fluid temperatures at the inlet (*T*_m,i_) and the exit. A differential pressure transducer was used to measure the pressure drop across the test section. The flow rate was held in the range of 0.4 to 21 mL/min. With the measured temperatures, heat flux, and the flow rate, the local heat transfer coefficients were calculated as follows:(16)

where *T*_m_(*x*) and *h*(*x*) are the mean temperature of fluid and the local heat transfer coefficient. The mean temperature of fluid at any axial location is given by,(17)

where *P*, , and *C*_p _are the surface perimeter, the mass flow rate, and the heat capacity, respectively.

**Figure 9:**
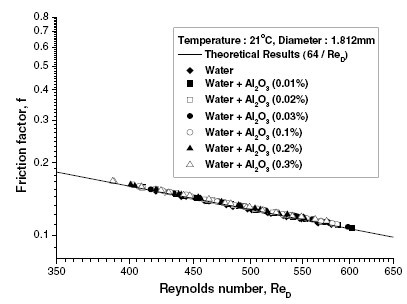
**Variation of the friction factor for water-based nanofluids in fully developed laminar flow, as given by Hwang et al. **[23].

The darcy friction factor for the flow of Al_2_O_3_-water nanofluids was calculated using the measured pressure drop in the pipe and plotted against the Reynolds number. The result was found to agree with the theoretical value for the fully developed laminar flow obtained from *f *= 64/Red, as shown in Figure 9. The measured heat transfer coefficient for water was found to provide an accuracy of measurement with less than 3% error when compared to the Shah equation. The convective heat transfer coefficient for nanofluids was found to be enhanced by around 8%, compared to pure water. It was proposed that the flattening of the fluid velocity profile in the presence of the nanoparticles could be one of the reasons for enhancement in the heat transfer coefficient.

## Convective heat transfer under constant wall-temperature condition

**Figure 10:**
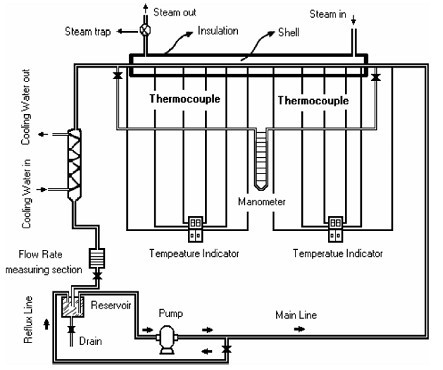
**Experimental setup of Heris et al. **[24].

Heris et al. [24] measured convective heat transfer in nanofluids in a circular tube, subjected to a constant wall temperature condition. The test section consisted of a concentric tube assembly of 1 m length. In this, the inner copper tube was of 6 mm diameter and 0.5 mm thickness, and the outer stainless steel tube was of 32 mm diameter, which was externally insulated with fiber glass. The experimental setup is shown schematically in Figure 10. The constant wall temperature condition was maintained by passing saturated steam through the annular section. The nanofluid flow rate was controlled by a reflux line with a valve. K-type thermocouples were used to measure the wall temperatures (*T*_w_) and bulk temperatures of the nanofluid at the inlet and the outlet (*T*_b1 _and *T*_b2_). A manometer was used to measure the pressure drop along the test section. From a measurement of the time required to fill the glass vessel, the flow rate was calculated. The uncertainties associated with the measurement of the temperature and the flow rate measurements were found to be 1.0 and 2.0%, respectively. The convective heat transfer coefficient and the Nusselt number were calculated as follows:(18)(19)

where (*T*_w _- *T*_b_)_LM _is the logarithmic mean temperature difference, *A, D*, and *L *cross-sectional area, diameter, and heated length of the pipe and  is the average flow velocity. The uncertainties of the calculated heat transfer coefficient, pressure drop, Peclet number, Nusselt number, and Reynolds number were 3, 3, 3, 4, and 2.5%, respectively. The convective heat transfer coefficient was measured for nanofluids in the laminar flow regime at constant wall temperature condition, for the volume concentration in the range of 0.2 to 2.5%. The experimental results were compared with the Sieder-Tate correlation. Addition of nanoparticles showed a deviation from the values obtained by the correlation, which was particularly significant at higher values of the Peclet number. Typically, at a Peclet number of 6000, the heat transfer coefficient was found to be enhanced by 1.16 times for 0.2% concentration and 1.41 times for 2.5% concentration.

## Convective heat transfer in thermally developing region

**Figure 11:**
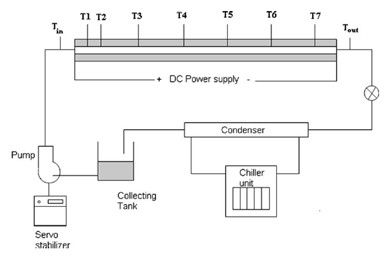
**Experimental setup of Anoop et al. **[25].

Anoop et al. [25] investigated the effect of the size of nanoparticles on forced convection heat transfer in nanofluids, focusing the study on the thermally developing region. The experimental forced circulation loop consisted of a pump, a heated test section (copper tube, 1200 mm length, 4.75 ± 0.05 mm inner diameter, 1.25 mm thickness), a cooling section, and a collecting tank, as shown in Figure 11. A constant laminar flow rate was maintained in the loop. A variable transformer connected to the electric circuit of the pump was used to vary the flow rates. The DC power source connected to the electrically insulated Ni-Cr wire, uniformly wound around the pipe dissipated a maximum power of 200 W. T-type thermocouples were used to measure the wall temperatures as well as the fluid inlet and exit temperatures.

Plug flow was maintained at the entrance using a series of wire meshes. A precise measuring jar and stop watch is used to measure the flow rates. The local heat transfer coefficient and local Nusselt number are defined by Equations 16, 17, and 19. The thermal conductivity value used was at the bulk mean temperature. The density and specific heat of the nanofluid dependent on the volume fraction, φ, was given by,(20)(21)

**Figure 12:**
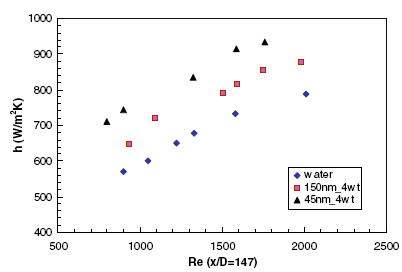
**Variation of heat transfer coefficient with particle size and Reynolds number as given by Anoop et al. **[25].

The convective heat transfer coefficient was measured with nanofluids mixed with Al_2_O_3 _nanoparticles of average sizes 45 and 150 nm. In the developing flow region and for a Reynolds number of 1500, the 45-nm sized particles gave 25% enhancement in heat transfer compared with 11% by the 150-nm particles, for a concentration of 4% by weight, as shown in Figure 12. The enhancement in heat transfer coefficient was also found to decrease, from the developing to fully developed region. For a concentration of 4% (by weight) of 45 nm particles and an approximate Reynolds number of 1500, the enhancement in heat transfer coefficient was 31% at *x*/*D *= 63, while it was 10% at *x*/*D *= 244. The uncertainty in the measurement of thermal conductivity was found to be less than 2%, and that for viscosity was 0.5%. A systematic uncertainty analysis yielded the maximum error in the Reynolds number and the Nusselt number to be around 3.24 and 2.45%, respectively.

## Single-phase and two-phase heat transfer in microchannels

**Figure 13:**
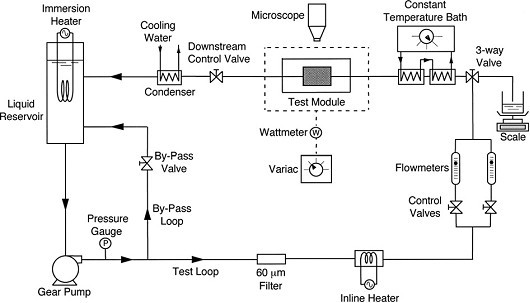
**Experimental setup of Lee et al. **[26].

Lee et al. [26] investigated on the use of nanofluids for single-phase and two-phase heat transfer in microchannels. The experimental setup used for the measurements is shown in Figure 13. The channels were fabricated by milling rectangular grooves, 215 μm wide and 821 μm deep, into the top surface of an oxygen-free copper block. The block was inserted into a G-7 plastic housing and sealed on top with a polycarbonate cover plate. The method produced 21 parallel microchannels, each with a hydraulic diameter of 341 μm, occupying a total substrate area with 1 cm width and 4.48 cm length. Heating was provided by 12 cartridge heaters embedded in the bottom of the copper block. The fluid temperature and pressure were measured at the inlet and exit plenums of the housing. The bottom wall temperature was also measured using K-type thermocouples inserted along the flow direction. A Yokogawa WT210 power meter was used to measure the electric power input to the copper block. A bypass was included immediately downstream of the flow-meters to calibrate the flow meters. An HP 3852A data acquisition system was utilized in the setup. Heat loss from the copper block was estimated as less than 5% of the electrical power input. The single phase flow experiments in the laminar regime showed an enhancement in heat transfer with the nanoparticle concentration. The fluid and pipe wall temperatures were found to increase with the nanoparticle concentration, which was interpreted as due to the reduced specific heat of nanofluids. The enhancement in heat transfer was found to be lesser in the turbulent flow regime than in the laminar regime. In the case of two phase heat transfer using nanofluids, it was observed that the chances of particles separating, getting deposited as clusters and thus clogging passages in micro-channels could make the method less preferable.

## Convective heat transfer in confined laminar radial flows

**Figure 14:**
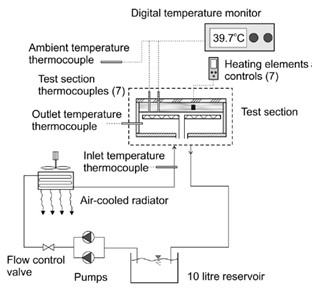
**Experimental setup of Gherasim et al. **[27].

Impinging jets with or without confinement as well as fluid flow between fixed or rotating disks with axial injection have applications in turbomachinery and localized cooling. Gherasim at al. [27] experimentally investigated the heat transfer enhancement capabilities of coolants with Al_2_O_3 _nanoparticles suspended in water inside a radial flow cooling system. The test rig was as shown in Figure 14. Parametric studies were performed on heat transfer inside the space delimited by the nozzle and the heated disk (Aluminum, 30 cm diameter, 7.5 cm thick), with and adjustable separating distance between them. The disk was heated with seven symmetrically implanted 200 W cartridge heating elements, one at the center of the disk, and the other six spaced at 60° from each other at approximately half the radial distance. Thermally insulated K-type thermocouples were used to measure the temperatures. The heated disk was insulated using a 1.5-cm Teflon disk and a 3-cm thick insulating foam board. The periphery of the test section was surrounded by insulating foam. The concentric inlet and outlet tubes were insulated from each other using a plastic sleeve and a layer of air. From the time required to accumulate a certain quantity of fluid, the fluid mass flow rate was calculated. The heat flux was varied by changing the tension applied to the heating elements. The applied power was calculated from the measured voltage and current. The Reynolds number, as defined in Equation 22, and the Nusselt number (Equation 19) were calculated:(22)

where  is the mean inlet fluid velocity and *D*_h _is given by 2δ, where *δ *is the distance separating the disks. The local heat transfer coefficient *h*_r _is obtained as:(23)

The bulk temperature at a given radial section (*T*_b,r_) was calculated as:(24)

where *T*_b,r _and *T*_b,i _are the bulk temperatures at a given radius and at the inlet.

Considering all the uncertainties on experimental measurements, the average relative errors on Nusselt number calculations were estimated as 12.1, 11.5, and 11% for cases with particle volume concentrations of 2, 4, and 6%, respectively. The experiments were aimed at investigating the effect of nanofluids in a steady laminar flow between the disk and a flat plate, with axial entry and radial exit. The heat transfer coefficient was found to increase with the particle concentration and the flow rate and decrease with an increasing gap between disks.

## Summary

A review of the important investigations on forced convection heat transfer in nanofluids, presented above reveals the following general inferences. Though not extensively, attention has been devoted to explore the fluid dynamic and thermal performance of nanofuids under various physical situations. Convective heat transfer studies have been carried out in the developing region [25,34] as well as under fully developed conditions [15]. Studies have been reported pertaining to laminar [23,24,27-29], transition [32,35], and turbulent [28,33] regimes of flow. Single phase and two phase flows have been analyzed with axial and radial flow directions [27]. Constant heat flux [25,28] and constant temperature [24,29] boundary conditions have been investigated. Studies have also been reported on flow and heat transfer in compact passages such as micro channels [26,30]. A comparison of the convective heat transfer coefficients for different nanofluids at various flow and heat transfer conditions reported in the literature [23-35] is shown in Table 3.

**Table 3:** Convective heat transfer coefficient and frictional effects

**Sl. no**.	Reference	Nanoparticle	Base fluid	Flow regime	Wall boumdary condition	Concentration	Enhancement in heat transfer coefficient	Pressure drop/friction factor
1	Hwang et al. [23]	Al_2_O_3 _(30 ± 5 nm)	Water	Fully developed laminar flow with	Constant heat flux	0.01-0.3 vol.%	@ *Re *= 700 for 0.3%, heat transfer coeff., *h *increases by 8%	Friction factor follows *f *= 64/*Re*_D_
2	Heris et al. [24]	Al_2_O_3_	Water	Laminar, *Re*:700-2050	Constant wall temp.	0.2, 0.5, 1.0, 1.5, 2.0, 2.5% volume	@ Peclet no., *Pe *= 6000 for 2.5%, *h *increases by 41%	Δ*P *= 200 Pa/m @ *Re *= 700 Δ*P *= 700 Pa/m @ *Re *= 2000
3	Anoop et al. [25]	Al_2_O_3 _(45 and 150 nm)	Water	Laminar thermally developing flow	Constant heat flux	1, 2, 4, and 6 wt%	@ *x*/*D *= 147, *Re *= 1550 and 4%, for 45 nm *h *increases by 25% and for 150 nm *h *increases by 11%	-
4	Lee et al. [26]	Al_2_O_3 _(36 nm)	Water	Laminar flow in microchannels, *Re*_Dh _= 140-941	Constant heat flux	1, 2% by volume	@ *Q *= 300 W, *Re *= 800 for 2%, *h *increases by 17%	@ *Re *= 800 Δ*P *= 21000 Pa for 2 vol.% ΔP = 15000 Pa for water.
5	Gherasim et al. [27]	Al_2_O_3 _(47 nm)	Water	Laminar radial flow	Constant heat flux	2, 4, and 6% by volume	@*q*" = 3900 W/m^2^, disk spacing of 2 mm and Re = 500 for 4%, heat transfer is doubled	-
6	Kim et al. [28]	Al_2_O_3 _(20-50 nm), amorphous carbonic nanofluids (20 nm)	Water	Laminar and turbulent flows	Constant heat flux	Amorphous carbonic nanofluids @3.5 vol.%, Al_2_O_3 _nanofluids @3 vol.%.	@*x*/*D *= 50, *Re *= 1460 for 3% Al_2_O_3_, *h *increases by 25% @*x*/*D *= 50, Re = 6020 for 3% Al_2_O_3_, *h *increases by 15%	-
7	Heris et al. [29]	CuO (50-60 nm), Al_2_O_3 _(20 nm)	Water	Laminar flows	Constant wall temp.	0.2-3 vol.%	@*Pe *= 6500 for 3% Al_2_O_3 _*Nu *= 8.5 @*Pe *= 6500 for 3% CuO *Nu *= 8	-
8	Jung et al. [30]	Al_2_O_3 _(170 nm)	Water, Water-Ethylene glycol 50:50	Laminar flow in rectangular microchannel	Constant heat flux	0.6, 1.2, 1.8% by volume	@*x*/*D *= 0, *Re *= 284 for 1.8% in water, *h *increases by 40%. @*x*/*D *= 0, *Re *= 32 for 1.8% in water-EG, *h *increases by 14%.	Friction factors comparable with that of water
9	Ding et al. [31]	Titanate (20 nm), CNT, titanate nanotubes (*d *= 10 nm and *l *= 100 nm), nano diamond (2-50 nm)	Water	Thermally developing laminar and turbulent flow	Constant heat flux	0-4 vol.%	Heat transfer deteriorates for ethylene glycol-based titania and aqueous-based nano-diamond nanofluids. Water-CNT nanofluids give max enhancement	
10	Sharma et al. [32]	Al_2_O_3 _(47 nm)	Water	Hydrodynamically and thermally developed Transition flow.	Constant heat flux	0.02, 0.1% by volume	For 0.1% in the range of *Re *= 3500-8000 heat transfer enhanced by 14-24%	-
11	Duangthongsuk et al. [33]	TiO_2 _(21 nm)	Water	Turbulent flow, *Re*-4000-17000	Double pipe counter flow heat exchanger	0.2 vol.%	*h *increases by 6-11% for the flow range of *Re *= 4000-17000	Pressure drop and friction factor of the nanofluid are close to those of water
12	Ding et al. [34]	MWCNT	Water	Laminar flow	Cosntant heat flux	0.1, 0.25, and 0.5% by volume	@*x*/*D *= 150, *Re *= 1200 for 0.1% *h *increases by 150%	-
13	Yu et al. [35]	SiC (170 nm)	Water	*Re *= 3300-13000	Constant heat flux	3.7 vol.%	@*Re *= 10000 h is enhanced by 60%	The pumping power penalty for SiC-water is lesser than for Al_2_O_3_-water

Comparison of enhancement of heat transfer coefficient and frictional effects in various nanofluids.

Almost all of the above investigations have shown that the performance of nanofluids in forced convection heat transfer is better than that of the base fluid. However, there have been studies which reported deterioration in convective heat transfer in ethylene glycol based titanate nanofluids [31]. It generally is noticed that the percentage enhancement in heat transfer is much more than the individual enhancement in thermal conductivity. This fact is often attributed to the effect of the disruption of the thermal boundary layer due to particle movement [25].

The enhancement of heat transfer capabilities of fluids results in accomplishing higher heat transfer rates without incorporating any modifications to existing heat exchangers. It also effectively leads to a reduction in the pumping power requirements in practical applications, as a lower flow rate will produce the required amount of heat transfer. These, in general makes the use of nanofluids for forced circulation loops attractive, leading to better performance and the resulting advantage in energy efficiency.

## Natural convection loops using nanofluids

Many of the investigations on natural convection phenomena in nanofluids deal with stagnant columns of the liquid, and in these studies, a possibility of reduction of the heat transfer coefficient has been observed [36]. Some investigators have discussed on the reasons for this behavior, and have suggested that this may be due to the reduction in the gradients of temperature within the fluid, resulting from the enhancement of the fluid thermal conductivity. However, natural circulation loops present a different scenario compared to convection in liquid columns, as the circulation is developed due to thermosyphon effect. It is of interest to look into some of the investigations on natural circulation loops with nanofluids, and understand the heat transfer performance under the influence of the nanoparticles. A few important articles on this topic are reviewed below. Some investigations on natural convection in stagnant fluid columns and pool boiling heat transfer are also reviewed.

**Figure 15:**
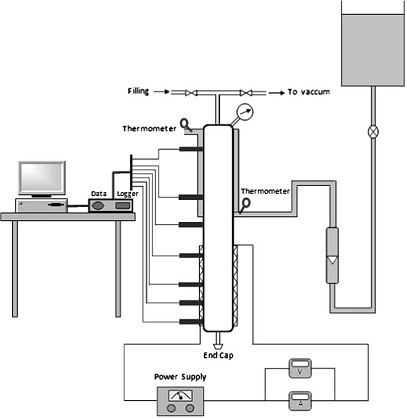
**Experimental setup of Noie et al. **[37].

Noie et al. [37] reported an enhancement in heat transfer when nanofluids were used in a two-phase closed thermosyphon (TPCT). The TPCT was made of a copper tube (20 mm internal diameter, 1 mm thick, 1000 mm long) and, the evaporator (300 mm long) and condenser (400 mm long) sections. Heating was provided by a Nickel-Chrome wire electric heater wound around the evaporator section, with a nominal power of 1000 W. The experimental setup was as shown in Figure 15.

The input power is given by:(25)

where *Q*_loss _is the total heat loss from the evaporator section by radiation and free convection:(26)

The radiation and free convection heat transfer rates were calculated as follows:(27)(28)

In the above, the free convection heat transfer coefficient was determined using the expression:(29)

The total heat loss was estimated to be about 2.49% of the input power to the evaporator section. As shown in Figure 15, LM35 thermocouples were mounted on the TPCT, evaporator section, adiabatic section, and condenser section. Precise thermometers were used in the condenser section to read the input and output temperature of the coolant water. All the measured data were monitored using a data acquisition system. The quantity of heat transferred to the coolant water was calculated as:(30)

The efficiency of the TPCT was expressed as a ratio of the output heat by condensation to the input heat by evaporation:(31)

**Figure 16:**
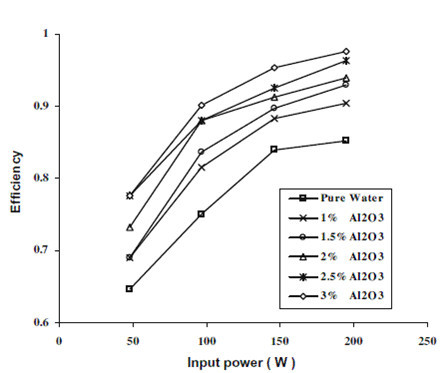
**Variation of efficiency of TPCT with nanoparticle concentration and input power as given by Noie et al. **[37].

Considering the measurement errors of the parameters such as the current, the voltage, the inlet and outlet temperature of cooling water, and the mass flow rate, and neglecting the effect of *Q*_loss_, the maximum uncertainty of the efficiency was calculated as 5.41%. Figure 16 shows that the efficiency of TPCT increases with nanoparticle concentration at all input power. For an input power of 97.1 W, the 1% nanofluid gives an efficiency of 85.6% as compared to 75.1% given by pure water.

**Figure 17:**
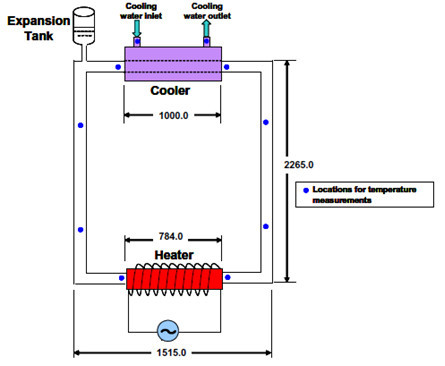
**Experimental setup of Nayak et al. **[38].

Nayak et al. [38] investigated the single phase natural circulation behavior of nanofluids in a rectangular loop. The test facility was made of glass tubes with 26.9 mm inner diameter, and had a heating section at the bottom and a cooling section at the top, as shown in Figure 17. The volumetric expansion of the fluid was accommodated by the expansion tank which also ensured that the loop remains full of water. Thermocouples were used to measure the instantaneous local temperature, and a pressure transducer installed in the horizontal leg of the loop measured the flow rate. The loop was insulated to minimize the heat losses to the ambient. The measurement accuracy was 0.4% (+1.1°C) for the thermocouples, +0.25% for the flow rate measurement and +0.5% of the range (0 to 1250 W) for power and pressure drop (-100 to +100 Pa). Experimental results have shown that the steady-state flow rate of nanofluids in the thermosyphon loop is higher compared to pure water. The flow rate is increased by 20 to 35% depending on the nanoparticle concentration and the heat input.

**Figure 18:**
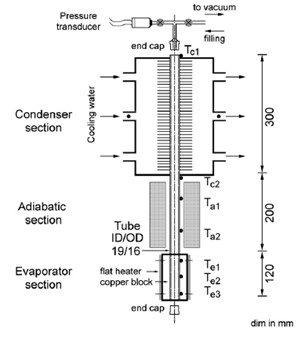
**Experimental setup of Khandekar et al. **[39].

Khandekar et al. [39] reported investigations on the thermal performance of a closed two-phase thermosyphon system, using pure water and various water-based nanofluids of Al_2_O_3_, CuO, and laponite clay as working fluids. The setup shown in Figure 18 has a pressure transducer fitted to the thermosyphon to monitor proper initial vacuum level and subsequent saturation pressure profiles. Four mica insulated surface heaters (116 mm × 48 mm) were mounted on the outer surface of a copper heating block (120 mm × 50 mm × 50 mm) with a center bore to accommodate the thermosyphon container which acts as the evaporator. The finned tube condenser was made of 40 square copper fins (70 mm × 70 mm × 1 mm), brazed at a pitch of 6.5 mm. The inlet and outlet of the shell side were designed so as to produce cross-flow conditions over the condenser fins. K-type thermocouples were used to measure the temperature at important axial locations on the thermosyphon tube. A PC based data acquisition system (NI-PCI-4351, National Instruments) was used to acquire the data.

The thermal resistance is defined as:(32)

where *T*_e _and *T*_c _are average values of the temperatures measured by the thermocouples.

The basic mechanisms of heat transfer, in a gravity-assisted thermosyphon, are nucleate pool boiling in the evaporator and film-wise condensation in the condenser section [14]. The boiling and condensation heat transfer rates are influenced by the thermophysical properties of the working fluid and the characteristic features of the solid substrate. Major limitations of the gravity assisted thermosyphon are the dry-out limitation, counter current flow limitation (CCFL) or flooding, and the boiling limitation. It was noticed that if the filling ratio (FR) is more than 40%, dry out phenomenon is not observed and the maximum heat flux is limited by the CCFL/flooding or the boiling limitation (BL).

The thermal performance of the system was found to be deteriorating when nanofluids were used as working fluids. The deterioration was maximum with laponite and minimum for aluminum oxide suspended nanofluids. Increased thermal conductivity of the nanofluids showed no effect on the nucleate pool boiling heat transfer coefficient. It was suggested that physical interaction of nanoparticles with the nucleating cavities has been influencing the boiling characteristics of the nanofluids. The deterioration of the thermal performance of the nanofluid in closed two-phase thermosyphon was attributed to the improvement in wettability due to entrapment of nanoparticles in the grooves present on the surface. Improved critical heat flux values were also observed, which effect was also attributed to the increased wettability characteristics of nanofluids.

Natural convection heat transfer is a preferred mode as it is comparatively noise less and does not have pumping power requirement. The use of Al_2_O_3_/water nanofluids in closed two-phase thermosyphon systems [37] has shown to increase its efficiency by 14.7% when compared to water. In rectangular loops [38] with water-based nanofluids, the flow instabilities were found to decrease and the circulation rates improved, compared to the base fluid. At the same time, there have been observations [39] that in two-phase thermosyphon loops, water-based nanofluids with suspended metal oxides have inferior thermal performance compared to the base fluids, which was explained as due to the increased surface wettability of nanofluids.

## Studies in stagnant columns

Experimental investigations have been reported on natural convection in stagnant columns, as well as pool boiling heat transfer in nanofluids. Measurement of critical heat flux (CHF) has also been reported in pool boiling.

Putra et al. [40] experimentally investigated the natural convection inside a horizontal cylinder heated from one side and cooled from the other. The effects of the particle concentration, the material of the particles, and the geometry of the containing cavity on natural convection were investigated. A systematic and definite deterioration of natural convection was observed and the deterioration increased with particle concentration. Copper oxide nanofluids showed larger deterioration than aluminum oxide nanofluids. With 4% Al_2_O_3 _concentration, an *L*/*D *ratio of 1.5 showed a higher value of Nusselt number compared to an *L*/*D *ratio of 0.5.

Liu et al. [41] studied the boiling heat transfer characteristics of nanofluids in a flat heat pipe evaporator with a micro-grooved heating surface. The nucleate boiling heat transfer coefficient and CHF of water-CuO nanofluids at different operating pressures and particle concentrations were measured. For a nanoparticle mass concentration less than 1%, the heat transfer coefficient and CHF were found to increase. Above 1% by weight, the CHF was almost constant and the heat transfer coefficient deteriorated. This was explained to be due to a decrease in the surface roughness and the solid-liquid contact angle. Heat transfer coefficient and CHF of nanofluids were found to increase with a reduction in the pressure. At the atmospheric pressure, the heat transfer coefficient and CHF showed 25 and 50% enhancement, respectively, compared to 150 and 200% enhancement at a pressure of 7.4 kPa.

Boiling heat transfer on a high-temperature silver sphere immersed in TiO_2 _nanofluid was investigated by Lotfi et al. [42]. A 10 mm diameter silver sphere heated to 700°C was immersed in the nanofluid at 90°C to study the boiling heat transfer and quenching capabilities. Film boiling heat flux in the TiO_2 _nanofluid was found to be lower than that in water. The accumulation of nanoparticles at the liquid-vapor interface was found to reduce the vapor removal rate from the film, creating a thick vapor film barrier which reduced the minimum film boiling heat flux. Experiments by Narayan et al. [43] showed that surface orientation has an influence on pool boiling heat transfer in nanoparticle suspensions. A smooth heated tube was suspended at different orientations in nanofluids to study the pool boiling performance. The pool boiling heat transfer was found to be maximum for the horizontal inclination. Al_2_O_3_-water nanofluids of 47 nm particles and 1% by weight concentration showed enhancement in pool boiling heat transfer performance, over that of water. With increase in concentration and particle size, the performance decreased for nanofluids. For vertical and 45° inclination orientations, nanofluids showed inferior performance compared to pure water. Coursey and Kim [44] investigated the effect of surface wettability on the boiling performance of nanofluids. In the experiments, heater surfaces altered to varying degrees by oxidization or by depositing metal were investigated by measuring the surface energy measurements and boiling heat transfer (CHF). It was found that the CHF of poorly wetting systems could be improved by up to 37% by the use of nanofluids, while surfaces with good wetting characteristics showed less improvement.

## Conclusion

Suspending nanoparticles in base fluids has proven to show considerable effects on various thermophysical properties, which influences the heat transfer performance. This article focused on some of the recently reported investigations on convective heat transfer and phase change in nanofluids. It also presented some discussions on the experimental techniques employed to measure the effective thermal conductivity, as well as to characterize the thermal performance of systems involving nanofluids.

The thermal conductivity of nanofluids has been measured using transient and steady-state methods, of which the transient hot wire method is found to be more versatile, accurate, and reliable. A review of the important investigations on forced convection heat transfer at various flow and heat transfer conditions have shown that the performance of nanofluids in forced convection is better than that of the base fluid. It has also been noticed that the percentage enhancement in heat transfer is much more than the individual enhancement in thermal conductivity.

The use of nanofluids in thermosyphon loops has shown an increase in the efficiency, a decrease in flow instabilities, and an increase in the flow rates. There have also been observations that in two-phase thermosyphon loops, the increased wettability of nanofluids may adversely affect the thermal performance compared to that of the base fluid.

Investigation on the natural convection inside a horizontal cylinder heated from one side and cooled from the other has shown deterioration in heat transfer while nanofluids are used. At low nanoparticle mass concentrations, the CHF was found to increase in a flat heat pipe. In pool boiling heat transfer in nanoparticle suspensions, the orientation of the heater surface is found to have an influence on the heat transfer rate, the maximum being for horizontal orientation. It has been noticed that for poorly wetting surfaces, the CHF can be increased by the use of nanofluids.

Of the various applications proposed, the use of nanofluids in closed circulation loops for sensible heat removal is found to be the most attractive, and these can become part of steady-state heat exchange systems. The enhancement of the heat transfer capability of fluids with suspended nanoparticles makes their use in convection loops and thermosyphons an interesting option, leading to better system performance and the resulting advantage in energy efficiency.

## Abbreviations

BL: boiling limitation; CCFL: counter current flow limitation; CHF: critical heat flux; MEMS: micro electro-mechanical systems; PDMS: polydimethylsiloxane; PECVD: plasma enhanced chemical vapor deposition; POM: polyoxymethylene; SHW: short hot wire; THS: transient hot strip; TPCT: two-phase closed thermosyphon.

## Competing interests

The authors declare that they have no competing interests.

## Authors' contributions

ST compiled the studies conducted on thermal conductivity, viscosity, free and forced convection and boiling phenomena, compared and analysed the results.

CBS contributed in conceptualizing the manuscript and revising it critically for improving technical contents.
